# Propane-1,2-diaminium tris­(pyridine-2,6-dicarboxyl­ato-κ^3^ *O* ^2^,*N*,*O* ^6^)zirconate(IV) trihydrate

**DOI:** 10.1107/S1600536811008488

**Published:** 2011-04-13

**Authors:** Hoda Pasdar, Shahrzad Shakiba, Hossein Aghabozorg, Behrouz Notash

**Affiliations:** a Department of Chemistry, Islamic Azad University, North Tehran Branch, Tehran, Iran; b Department of Chemistry, Shahid Beheshti University, G. C., Evin, Tehran 1983963113, Iran

## Abstract

In the title compound, (C_3_H_12_N_2_)[Zr(C_7_H_3_NO_4_)_3_]·3H_2_O, the Zr^IV^ cation is chelated by three pyridine-2,6-dicarboxyl­ate anions in a distorted tricapped trigonal–prismatic environment. The crystal structure is stabilized by inter­molecular N—H⋯O and O—H⋯O hydrogen bonds.

## Related literature

For the background to proton-transfer compounds, see: Aghabozorg *et al.* (2008 ▶). For related structures, see: Aghabozorg *et al.* (2005 ▶); Daneshvar *et al.* (2008 ▶); Pasdar *et al.* (2010**a* ▶,b* ▶, 2011**a* ▶,b* ▶).
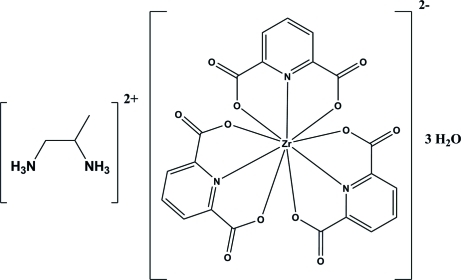


## Experimental

### 

#### Crystal data

(C_3_H_12_N_2_)[Zr(C_7_H_3_NO_4_)_3_]·3H_2_O
*M* *_r_* = 716.73
Monoclinic, 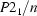

*a* = 10.515 (2) Å
*b* = 19.195 (4) Å
*c* = 14.149 (3) Å
β = 103.39 (3)°
*V* = 2778.1 (10) Å^3^
*Z* = 4
Mo *K*α radiation
μ = 0.48 mm^−1^
*T* = 298 K
0.25 × 0.15 × 0.15 mm

#### Data collection

Stoe IPDS II diffractometer
Absorption correction: numerical (*X-SHAPE* and *X-RED32*; Stoe & Cie, 2005 ▶) *T*_min_ = 0.915, *T*_max_ = 0.926
21838 measured reflections
7481 independent reflections
5264 reflections with *I* > 2σ(*I*)
*R*_int_ = 0.133

#### Refinement

*R*[*F* ^2^ > 2σ(*F* ^2^)] = 0.086
*wR*(*F* ^2^) = 0.206
*S* = 1.18
7481 reflections
427 parameters
7 restraints
H atoms treated by a mixture of independent and constrained refinement
Δρ_max_ = 1.09 e Å^−3^
Δρ_min_ = −0.77 e Å^−3^

### 

Data collection: *X-AREA* (Stoe & Cie, 2005 ▶); cell refinement: *X-AREA* (Stoe & Cie, 2005 ▶); data reduction: *X-AREA*; program(s) used to solve structure: *SHELXS97* (Sheldrick, 2008 ▶); program(s) used to refine structure: *SHELXL97* (Sheldrick, 2008 ▶); molecular graphics: *ORTEP-3 for Windows* (Farrugia, 1997 ▶); software used to prepare material for publication: *WinGX* (Farrugia, 1999 ▶).

## Supplementary Material

Crystal structure: contains datablocks I, global. DOI: 10.1107/S1600536811008488/xu5157sup1.cif

Structure factors: contains datablocks I. DOI: 10.1107/S1600536811008488/xu5157Isup2.hkl

Additional supplementary materials:  crystallographic information; 3D view; checkCIF report

## Figures and Tables

**Table 1:** Hydrogen-bond geometry (Å, °)

*D*—H⋯*A*	*D*—H	H⋯*A*	*D*⋯*A*	*D*—H⋯*A*
O13—H13*A*⋯O14	0.80 (8)	2.10 (9)	2.745 (11)	138 (13)
O13—H13*B*⋯O2^i^	0.80 (4)	2.10 (8)	2.793 (8)	145 (12)
O14—H14*A*⋯O5^ii^	0.91 (8)	2.11 (9)	2.978 (7)	158 (12)
O14—H14*B*⋯O8	0.85 (4)	1.89 (5)	2.731 (9)	171 (14)
O15—H15*A*⋯O12	0.98 (9)	1.91 (10)	2.857 (8)	164 (12)
O15—H15*B*⋯O1^iii^	0.83 (4)	2.46 (7)	3.227 (9)	155 (13)
N4—H4*A*⋯O10^iv^	0.89	1.92	2.791 (8)	165
N4—H4*B*⋯O6^ii^	0.89	2.24	2.851 (7)	125
N4—H4*C*⋯O15^v^	0.89	2.04	2.864 (10)	154
N5—H5*A*⋯O13	0.89	1.94	2.769 (9)	154
N5—H5*B*⋯O6^iv^	0.89	2.18	2.844 (8)	131
N5—H5*C*⋯O4	0.89	1.91	2.776 (8)	163

Symmetry codes: (i) 
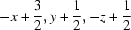
; (ii) 
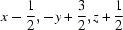
; (iii) 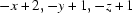; (iv) 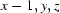; (v) 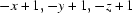.

**Fig. 1.**
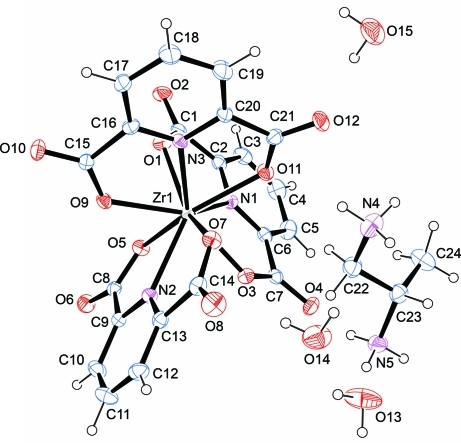
The molecular structure of the title compound with displacement ellipsoids drawn at 30% probability level.

**Fig. 2.**
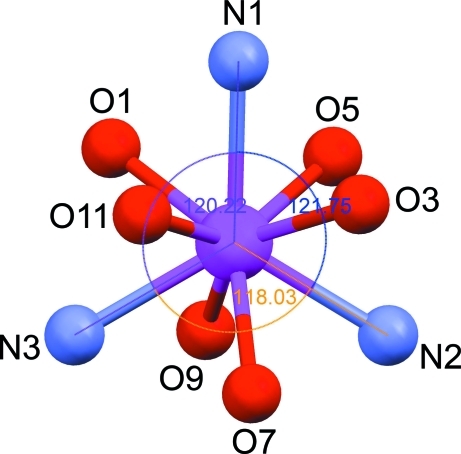
The coordination environment around ZrIV ion in the title compound.

**Fig. 3.**
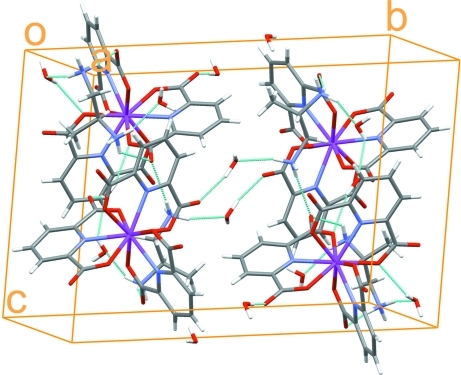
The packing diagram of the title compound. The intermolecular N—H···O, O—H···O hydrogen bonds are shown as dashed lines.

## supplementary crystallographic information

### Comment

Pyridine-2,6-dicarboxylic acid (pydcH_2_) was commonly used by Aghabozorg and his co-workers as an acid in proton transfer systems (Aghabozorg *et al.* 2008). Our group have been focused on forming ion pairs between 2,6-pydcH_2_ and various organic bases (Pasdar *et al.*, 2010*a*; Pasdar *et al.* 2011**a*,b*). The structure of two proton transfer compound containing nine-coordinated [Zr^IV^(2,6-pydc)_3_]^2-^ moiety were reported with the counter cationic part of 2,6-pyridinediamine(Aghabozorg *et al.* 2005) and 2,4,6-triamino-1,3,5-triazine (Daneshvar *et al.* 2008), respectively. Recently, we report the structure of (2a6mpH)_2_[Zr(2,6-pydc)_3_].2H_2_O (Pasdar *et al.* 2011*a*)*.*

Herein, we report the synthesis and crystal structure of (1,2-pdaH_2_)[Zr(2,6-pydc)_3_].3H_2_O. The title compound was prepared by the reaction of ZrCl_4_.3H_2_O, propane-1,2-diamine and 2,6-pyridinedicarboxylic acid in aqueous solution. Fig.1 present the molecular structure of the title compound. X-ray diffraction study shows that Zirconium(IV) ion is coordinated by three pydc^2-^ moiety in a distorted tricapped trigonal prismatic geometry and pydc^2-^ ligands act as tridentate ligand. Coordination environment around Zirconium(IV) ion in the (1,2-pdaH_2_)[Zr(2,6-pydc)_3_].3H_2_O is presented in Fig. 2. The Zr—N and Zr—O bond lengths and angles are in the normal ranges (Aghabozorg *et al.* 2005; Daneshvar *et al.* 2008). The crystal packing diagram of the title compound is shown in Fig. 3. In the crystal packing diagram of the title compound, there are several intermolecular N—H···O, O—H···O hydrogen bonds which play an important role in stabilization of crystal structure (Table 1 and Fig. 3).

### Experimental

A solution of propane-1,2-diamine (0.074 g, 1 mmol) in water (8 ml) and 2,6-pyridinedicarboxylic acid (0.501 g, 3 mmol) in water (10 ml) were mixed and stirred until clear solution obtained. Then a solution of ZrCl_4_.3H_2_O (0.116 g, 0.5 mmol) in water (5 ml) was added to the mixture of acid-base and stirrer for 5 h. Crystals of the title compound suitable for X-ray analysis were obtained by slow evaporation after one month at room temperature (m.p: 210 °C).

### Refinement

Water H atoms were found in a difference Fourier map and positional parameters were refined, *U*_iso_(H) = 0.115 Å^2^. Ammonium H atoms were positioned geometrically and refined as riding atoms with N—H = 0.89 Å and *U*_iso_(H) = 1.5*U*_eq_(N). Other H atoms were positioned geometrically and refined as riding atoms with C—H = 0.93-0.98 Å, *U*iso(H) = 1.5*U*eq(C) for methyl H atoms and 1.2*Ueq*(C) for the others.

### Crystal data

**Table:**

(C_3_H_12_N_2_)[Zr(C_7_H_3_NO_4_)_3_]·3H_2_O	*F*(000) = 1464
*M**_r_* = 716.73	*D*_x_ = 1.714 Mg m^−^^3^
Monoclinic, *P*2_1_/*n*	Mo *K*α radiation, λ = 0.71073 Å
Hall symbol: -P 2yn	Cell parameters from 7481 reflections
*a* = 10.515 (2) Å	θ = 2.1–29.2°
*b* = 19.195 (4) Å	µ = 0.48 mm^−^^1^
*c* = 14.149 (3) Å	*T* = 298 K
β = 103.39 (3)°	Prism, colorless
*V* = 2778.1 (10) Å^3^	0.25 × 0.15 × 0.15 mm
*Z* = 4	

### Data collection

**Table:**

Stoe IPDS II diffractometer	7481 independent reflections
Radiation source: fine-focus sealed tube	5264 reflections with *I* > 2σ(*I*)
graphite	*R*_int_ = 0.133
rotation method scans	θ_max_ = 29.2°, θ_min_ = 2.1°
Absorption correction: numerical (*X-SHAPE* and *X-RED32*; Stoe & Cie, 2005)	*h* = −14→11
*T*_min_ = 0.915, *T*_max_ = 0.926	*k* = −26→26
21838 measured reflections	*l* = −19→19

### Refinement

**Table:**

Refinement on *F*^2^	Primary atom site location: structure-invariant direct methods
Least-squares matrix: full	Secondary atom site location: difference Fourier map
*R*[*F*^2^ > 2σ(*F*^2^)] = 0.086	Hydrogen site location: inferred from neighbouring sites
*wR*(*F*^2^) = 0.206	H atoms treated by a mixture of independent and constrained refinement
*S* = 1.18	*w* = 1/[σ^2^(*F*_o_^2^) + (0.0753*P*)^2^ + 3.9962*P*] where *P* = (*F*_o_^2^ + 2*F*_c_^2^)/3
7481 reflections	(Δ/σ)_max_ = 0.001
427 parameters	Δρ_max_ = 1.09 e Å^−^^3^
7 restraints	Δρ_min_ = −0.77 e Å^−^^3^

### Special details

**Table:**

Geometry. All e.s.d.'s (except the e.s.d. in the dihedral angle between two l.s. planes) are estimated using the full covariance matrix. The cell e.s.d.'s are taken into account individually in the estimation of e.s.d.'s in distances, angles and torsion angles; correlations between e.s.d.'s in cell parameters are only used when they are defined by crystal symmetry. An approximate (isotropic) treatment of cell e.s.d.'s is used for estimating e.s.d.'s involving l.s. planes.
Refinement. Refinement of *F*^2^ against ALL reflections. The weighted *R*-factor *wR* and goodness of fit *S* are based on *F*^2^, conventional *R*-factors *R* are based on *F*, with *F* set to zero for negative *F*^2^. The threshold expression of *F*^2^ > σ(*F*^2^) is used only for calculating *R*-factors(gt) *etc*. and is not relevant to the choice of reflections for refinement. *R*-factors based on *F*^2^ are statistically about twice as large as those based on *F*, and *R*- factors based on ALL data will be even larger.

### Fractional atomic coordinates and isotropic or equivalent isotropic displacement parameters (Å^2^)

**Table:**

	*x*	*y*	*z*	*U*_iso_*/*U*_eq_	
C18	1.0667 (8)	0.5828 (4)	0.6397 (5)	0.0486 (18)	
H18	1.0902	0.5663	0.7032	0.058*	
O13	0.3681 (10)	0.8559 (3)	0.2972 (5)	0.084 (2)	
O14	0.5141 (7)	0.8422 (4)	0.4841 (5)	0.0666 (18)	
Zr1	0.93403 (5)	0.68252 (3)	0.29848 (3)	0.02015 (14)	
O11	0.7886 (4)	0.6046 (2)	0.3278 (3)	0.0299 (9)	
O5	1.0314 (4)	0.7169 (2)	0.1812 (3)	0.0285 (8)	
O9	1.1276 (4)	0.7203 (2)	0.3786 (3)	0.0281 (8)	
O3	0.7472 (4)	0.7187 (2)	0.2025 (3)	0.0281 (8)	
N2	0.9428 (5)	0.8044 (2)	0.2844 (3)	0.0250 (9)	
O7	0.8366 (4)	0.7374 (2)	0.4015 (3)	0.0318 (9)	
O1	1.0691 (4)	0.5933 (2)	0.2947 (3)	0.0311 (9)	
N3	0.9968 (5)	0.6338 (2)	0.4535 (3)	0.0242 (9)	
N1	0.8636 (5)	0.6103 (2)	0.1615 (3)	0.0242 (9)	
O4	0.5741 (4)	0.7014 (3)	0.0797 (3)	0.0364 (10)	
C7	0.6845 (6)	0.6870 (3)	0.1263 (4)	0.0279 (11)	
C15	1.1900 (6)	0.6989 (3)	0.4611 (4)	0.0254 (11)	
O6	1.1211 (5)	0.7982 (3)	0.1045 (3)	0.0385 (10)	
O2	1.1365 (5)	0.5005 (2)	0.2250 (4)	0.0421 (11)	
O8	0.7646 (6)	0.8342 (3)	0.4614 (4)	0.0499 (13)	
C6	0.7542 (6)	0.6251 (3)	0.0975 (4)	0.0278 (11)	
C2	0.9348 (6)	0.5542 (3)	0.1484 (4)	0.0282 (11)	
C9	1.0020 (6)	0.8333 (3)	0.2204 (4)	0.0267 (11)	
C1	1.0562 (6)	0.5475 (3)	0.2271 (4)	0.0276 (11)	
C8	1.0568 (5)	0.7797 (3)	0.1625 (4)	0.0266 (11)	
C14	0.8231 (6)	0.8035 (3)	0.4075 (4)	0.0307 (12)	
C13	0.8855 (6)	0.8446 (3)	0.3394 (4)	0.0287 (12)	
O10	1.3031 (4)	0.7132 (3)	0.5023 (3)	0.0359 (10)	
O12	0.6980 (5)	0.5485 (3)	0.4335 (3)	0.0429 (11)	
C16	1.1115 (6)	0.6500 (3)	0.5102 (4)	0.0273 (11)	
C4	0.7813 (8)	0.5259 (4)	0.0028 (5)	0.0460 (17)	
H4	0.7531	0.4974	−0.0511	0.055*	
C20	0.9149 (6)	0.5919 (3)	0.4871 (4)	0.0283 (11)	
C21	0.7902 (6)	0.5797 (3)	0.4117 (4)	0.0308 (12)	
C3	0.8944 (7)	0.5103 (3)	0.0707 (5)	0.0375 (14)	
H3	0.9426	0.4707	0.0639	0.045*	
C11	0.9459 (8)	0.9464 (3)	0.2640 (5)	0.0440 (17)	
H11	0.9467	0.9946	0.2570	0.053*	
C5	0.7101 (7)	0.5843 (4)	0.0153 (5)	0.0406 (15)	
H5	0.6342	0.5961	−0.0303	0.049*	
C19	0.9465 (8)	0.5654 (4)	0.5800 (5)	0.0440 (16)	
H19	0.8887	0.5365	0.6022	0.053*	
C12	0.8857 (7)	0.9168 (3)	0.3310 (5)	0.0378 (14)	
H12	0.8460	0.9445	0.3698	0.045*	
C17	1.1515 (6)	0.6252 (4)	0.6036 (4)	0.0363 (14)	
H17	1.2335	0.6365	0.6417	0.044*	
C10	1.0048 (7)	0.9048 (3)	0.2074 (5)	0.0380 (14)	
H10	1.0453	0.9241	0.1617	0.046*	
N5	0.3816 (6)	0.7484 (3)	0.1693 (4)	0.0407 (13)	
H5A	0.3872	0.7903	0.1969	0.061*	
H5B	0.3091	0.7460	0.1222	0.061*	
H5C	0.4509	0.7413	0.1444	0.061*	
C23	0.3774 (7)	0.6935 (4)	0.2445 (5)	0.0379 (14)	
H23	0.3049	0.7040	0.2751	0.045*	
C22	0.5033 (7)	0.6975 (4)	0.3206 (5)	0.0419 (16)	
H22A	0.5725	0.6760	0.2958	0.050*	
H22B	0.5263	0.7460	0.3342	0.050*	
N4	0.4933 (6)	0.6623 (4)	0.4117 (4)	0.0493 (16)	
H4A	0.4389	0.6860	0.4394	0.074*	
H4B	0.5720	0.6605	0.4520	0.074*	
H4C	0.4631	0.6192	0.3983	0.074*	
C24	0.3509 (10)	0.6242 (4)	0.1930 (5)	0.053 (2)	
H24A	0.4143	0.6165	0.1549	0.080*	
H24B	0.2648	0.6246	0.1512	0.080*	
H24C	0.3569	0.5877	0.2401	0.080*	
O15	0.6503 (7)	0.4645 (4)	0.5884 (5)	0.077 (2)	
H15A	0.672 (13)	0.485 (7)	0.531 (7)	0.115*	
H14A	0.497 (14)	0.820 (7)	0.537 (8)	0.115*	
H14B	0.592 (6)	0.835 (7)	0.479 (10)	0.115*	
H13A	0.426 (11)	0.870 (7)	0.340 (8)	0.115*	
H13B	0.342 (13)	0.892 (4)	0.271 (8)	0.115*	
H15B	0.728 (6)	0.453 (8)	0.601 (10)	0.115*	

### Atomic displacement parameters (Å^2^)

**Table:**

	*U*^11^	*U*^22^	*U*^33^	*U*^12^	*U*^13^	*U*^23^
C18	0.053 (4)	0.059 (4)	0.029 (3)	−0.008 (4)	0.000 (3)	0.019 (3)
O13	0.141 (8)	0.037 (3)	0.070 (4)	0.017 (4)	0.017 (5)	−0.005 (3)
O14	0.072 (4)	0.072 (4)	0.066 (4)	0.018 (4)	0.037 (3)	0.026 (3)
Zr1	0.0211 (2)	0.0215 (2)	0.0184 (2)	0.0005 (2)	0.00570 (16)	0.0007 (2)
O11	0.032 (2)	0.032 (2)	0.0257 (18)	−0.0045 (17)	0.0058 (16)	0.0019 (16)
O5	0.035 (2)	0.027 (2)	0.0275 (19)	0.0021 (17)	0.0147 (17)	−0.0016 (15)
O9	0.029 (2)	0.028 (2)	0.0282 (19)	−0.0051 (16)	0.0097 (16)	0.0039 (15)
O3	0.030 (2)	0.029 (2)	0.0250 (18)	0.0030 (16)	0.0057 (16)	−0.0003 (15)
N2	0.026 (2)	0.024 (2)	0.027 (2)	0.0010 (18)	0.0093 (18)	−0.0020 (16)
O7	0.038 (2)	0.034 (2)	0.0275 (19)	0.0006 (18)	0.0161 (18)	0.0001 (17)
O1	0.037 (2)	0.031 (2)	0.0264 (19)	0.0058 (18)	0.0080 (17)	0.0005 (16)
N3	0.027 (2)	0.024 (2)	0.023 (2)	−0.0020 (18)	0.0079 (18)	0.0026 (17)
N1	0.026 (2)	0.025 (2)	0.023 (2)	0.0015 (18)	0.0070 (18)	0.0011 (17)
O4	0.024 (2)	0.047 (3)	0.037 (2)	0.0043 (18)	0.0039 (18)	0.0041 (19)
C7	0.030 (3)	0.028 (3)	0.027 (2)	−0.003 (2)	0.010 (2)	0.004 (2)
C15	0.025 (3)	0.024 (3)	0.027 (2)	0.001 (2)	0.007 (2)	−0.0047 (19)
O6	0.037 (2)	0.049 (3)	0.034 (2)	−0.001 (2)	0.0153 (19)	0.0118 (19)
O2	0.043 (3)	0.026 (2)	0.059 (3)	0.010 (2)	0.017 (2)	0.004 (2)
O8	0.056 (3)	0.053 (3)	0.050 (3)	0.007 (2)	0.030 (3)	−0.011 (2)
C6	0.032 (3)	0.028 (3)	0.027 (2)	−0.006 (2)	0.012 (2)	0.002 (2)
C2	0.030 (3)	0.027 (3)	0.031 (3)	−0.002 (2)	0.014 (2)	0.000 (2)
C9	0.023 (3)	0.026 (3)	0.030 (3)	0.000 (2)	0.004 (2)	0.003 (2)
C1	0.030 (3)	0.020 (2)	0.035 (3)	0.001 (2)	0.013 (2)	0.003 (2)
C8	0.023 (3)	0.033 (3)	0.023 (2)	0.001 (2)	0.003 (2)	0.006 (2)
C14	0.028 (3)	0.037 (3)	0.028 (3)	0.003 (2)	0.009 (2)	−0.006 (2)
C13	0.026 (3)	0.031 (3)	0.027 (3)	0.000 (2)	0.003 (2)	−0.005 (2)
O10	0.023 (2)	0.049 (3)	0.034 (2)	−0.0060 (19)	0.0039 (17)	0.001 (2)
O12	0.036 (2)	0.050 (3)	0.045 (2)	−0.011 (2)	0.013 (2)	0.016 (2)
C16	0.029 (3)	0.029 (3)	0.023 (2)	0.004 (2)	0.003 (2)	−0.001 (2)
C4	0.053 (4)	0.048 (4)	0.039 (3)	−0.011 (3)	0.014 (3)	−0.014 (3)
C20	0.028 (3)	0.030 (3)	0.026 (2)	−0.002 (2)	0.006 (2)	0.004 (2)
C21	0.028 (3)	0.033 (3)	0.031 (3)	0.004 (2)	0.007 (2)	0.003 (2)
C3	0.050 (4)	0.028 (3)	0.039 (3)	−0.002 (3)	0.019 (3)	−0.005 (2)
C11	0.063 (5)	0.024 (3)	0.045 (3)	−0.002 (3)	0.013 (3)	−0.003 (3)
C5	0.040 (4)	0.049 (4)	0.031 (3)	−0.004 (3)	0.003 (3)	−0.008 (3)
C19	0.047 (4)	0.050 (4)	0.036 (3)	−0.008 (3)	0.011 (3)	0.013 (3)
C12	0.041 (4)	0.029 (3)	0.040 (3)	0.007 (3)	0.002 (3)	−0.008 (3)
C17	0.031 (3)	0.041 (3)	0.032 (3)	−0.003 (3)	0.000 (2)	0.007 (3)
C10	0.044 (4)	0.032 (3)	0.037 (3)	−0.008 (3)	0.009 (3)	0.008 (2)
N5	0.042 (3)	0.038 (3)	0.043 (3)	0.004 (2)	0.012 (3)	0.009 (2)
C23	0.035 (3)	0.045 (4)	0.037 (3)	0.002 (3)	0.013 (3)	0.004 (3)
C22	0.037 (4)	0.049 (4)	0.040 (3)	−0.002 (3)	0.007 (3)	0.004 (3)
N4	0.044 (3)	0.072 (4)	0.032 (3)	0.008 (3)	0.010 (3)	0.001 (3)
C24	0.079 (6)	0.041 (4)	0.040 (4)	−0.013 (4)	0.013 (4)	0.001 (3)
O15	0.071 (4)	0.091 (5)	0.068 (4)	−0.024 (4)	0.014 (4)	0.031 (4)

### Geometric parameters (Å, °)

**Table:**

C18—C17	1.388 (10)	C9—C10	1.387 (8)
C18—C19	1.389 (10)	C9—C8	1.509 (8)
C18—H18	0.9300	C14—C13	1.508 (9)
O13—H13A	0.80 (8)	C13—C12	1.392 (9)
O13—H13B	0.80 (4)	O12—C21	1.239 (8)
O14—H14A	0.91 (8)	C16—C17	1.376 (8)
O14—H14B	0.85 (4)	C4—C3	1.378 (10)
Zr1—O9	2.210 (4)	C4—C5	1.382 (11)
Zr1—O3	2.225 (4)	C4—H4	0.9300
Zr1—O7	2.230 (4)	C20—C19	1.377 (8)
Zr1—O1	2.233 (4)	C20—C21	1.504 (8)
Zr1—O5	2.241 (4)	C3—H3	0.9300
Zr1—O11	2.245 (4)	C11—C10	1.375 (10)
Zr1—N3	2.334 (4)	C11—C12	1.379 (11)
Zr1—N2	2.352 (4)	C11—H11	0.9300
Zr1—N1	2.358 (4)	C5—H5	0.9300
O11—C21	1.277 (7)	C19—H19	0.9300
O5—C8	1.275 (7)	C12—H12	0.9300
O9—C15	1.267 (7)	C17—H17	0.9300
O3—C7	1.280 (7)	C10—H10	0.9300
N2—C9	1.333 (7)	N5—C23	1.506 (8)
N2—C13	1.334 (7)	N5—H5A	0.8900
O7—C14	1.283 (7)	N5—H5B	0.8900
O1—C1	1.283 (7)	N5—H5C	0.8900
N3—C16	1.321 (7)	C23—C22	1.502 (9)
N3—C20	1.343 (7)	C23—C24	1.510 (10)
N1—C6	1.320 (7)	C23—H23	0.9800
N1—C2	1.350 (7)	C22—N4	1.480 (9)
O4—C7	1.226 (7)	C22—H22A	0.9700
C7—C6	1.502 (8)	C22—H22B	0.9700
C15—O10	1.227 (7)	N4—H4A	0.8900
C15—C16	1.521 (8)	N4—H4B	0.8900
O6—C8	1.230 (7)	N4—H4C	0.8900
O2—C1	1.241 (7)	C24—H24A	0.9600
O8—C14	1.233 (8)	C24—H24B	0.9600
C6—C5	1.390 (8)	C24—H24C	0.9600
C2—C3	1.372 (8)	O15—H15A	0.98 (9)
C2—C1	1.492 (8)	O15—H15B	0.83 (4)
			
C17—C18—C19	119.2 (6)	O6—C8—O5	125.9 (6)
C17—C18—H18	120.4	O6—C8—C9	120.2 (5)
C19—C18—H18	120.4	O5—C8—C9	114.0 (5)
H13A—O13—H13B	100 (8)	O8—C14—O7	126.3 (6)
H14A—O14—H14B	111 (10)	O8—C14—C13	119.9 (6)
O9—Zr1—O3	142.08 (15)	O7—C14—C13	113.7 (5)
O9—Zr1—O7	91.02 (16)	N2—C13—C12	121.0 (6)
O3—Zr1—O7	77.11 (15)	N2—C13—C14	113.2 (5)
O9—Zr1—O1	75.43 (16)	C12—C13—C14	125.8 (6)
O3—Zr1—O1	134.53 (15)	N3—C16—C17	122.3 (6)
O7—Zr1—O1	140.25 (15)	N3—C16—C15	112.5 (5)
O9—Zr1—O5	76.03 (15)	C17—C16—C15	125.1 (5)
O3—Zr1—O5	86.94 (15)	C3—C4—C5	119.6 (6)
O7—Zr1—O5	134.60 (16)	C3—C4—H4	120.2
O1—Zr1—O5	78.81 (15)	C5—C4—H4	120.2
O9—Zr1—O11	135.19 (14)	N3—C20—C19	121.8 (6)
O3—Zr1—O11	77.16 (15)	N3—C20—C21	111.6 (5)
O7—Zr1—O11	76.04 (16)	C19—C20—C21	126.7 (6)
O1—Zr1—O11	87.57 (16)	O12—C21—O11	124.8 (6)
O5—Zr1—O11	141.28 (15)	O12—C21—C20	120.2 (5)
O9—Zr1—N3	67.84 (15)	O11—C21—C20	115.0 (5)
O3—Zr1—N3	135.72 (16)	C2—C3—C4	118.8 (6)
O7—Zr1—N3	69.39 (16)	C2—C3—H3	120.6
O1—Zr1—N3	70.89 (15)	C4—C3—H3	120.6
O5—Zr1—N3	137.31 (16)	C10—C11—C12	120.1 (6)
O11—Zr1—N3	67.44 (15)	C10—C11—H11	120.0
O9—Zr1—N2	70.58 (16)	C12—C11—H11	120.0
O3—Zr1—N2	71.60 (16)	C4—C5—C6	118.5 (6)
O7—Zr1—N2	67.56 (16)	C4—C5—H5	120.7
O1—Zr1—N2	136.30 (17)	C6—C5—H5	120.7
O5—Zr1—N2	67.09 (15)	C20—C19—C18	118.5 (6)
O11—Zr1—N2	136.09 (16)	C20—C19—H19	120.8
N3—Zr1—N2	118.01 (16)	C18—C19—H19	120.8
O9—Zr1—N1	133.70 (16)	C11—C12—C13	118.7 (6)
O3—Zr1—N1	67.48 (15)	C11—C12—H12	120.7
O7—Zr1—N1	135.26 (16)	C13—C12—H12	120.7
O1—Zr1—N1	67.05 (15)	C16—C17—C18	118.5 (6)
O5—Zr1—N1	71.04 (16)	C16—C17—H17	120.8
O11—Zr1—N1	70.27 (16)	C18—C17—H17	120.8
N3—Zr1—N1	120.25 (16)	C11—C10—C9	118.2 (6)
N2—Zr1—N1	121.74 (15)	C11—C10—H10	120.9
C21—O11—Zr1	123.9 (4)	C9—C10—H10	120.9
C8—O5—Zr1	125.9 (4)	C23—N5—H5A	109.5
C15—O9—Zr1	125.8 (4)	C23—N5—H5B	109.5
C7—O3—Zr1	125.1 (4)	H5A—N5—H5B	109.5
C9—N2—C13	120.1 (5)	C23—N5—H5C	109.5
C9—N2—Zr1	120.3 (4)	H5A—N5—H5C	109.5
C13—N2—Zr1	119.6 (4)	H5B—N5—H5C	109.5
C14—O7—Zr1	125.9 (4)	C22—C23—N5	107.8 (6)
C1—O1—Zr1	125.8 (4)	C22—C23—C24	115.1 (7)
C16—N3—C20	119.7 (5)	N5—C23—C24	107.9 (6)
C16—N3—Zr1	119.8 (4)	C22—C23—H23	108.6
C20—N3—Zr1	120.5 (4)	N5—C23—H23	108.6
C6—N1—C2	120.1 (5)	C24—C23—H23	108.6
C6—N1—Zr1	119.7 (4)	N4—C22—C23	111.8 (6)
C2—N1—Zr1	120.2 (4)	N4—C22—H22A	109.3
O4—C7—O3	125.8 (6)	C23—C22—H22A	109.3
O4—C7—C6	119.5 (5)	N4—C22—H22B	109.3
O3—C7—C6	114.6 (5)	C23—C22—H22B	109.3
O10—C15—O9	127.1 (6)	H22A—C22—H22B	107.9
O10—C15—C16	119.3 (5)	C22—N4—H4A	109.5
O9—C15—C16	113.6 (5)	C22—N4—H4B	109.5
N1—C6—C5	121.5 (6)	H4A—N4—H4B	109.5
N1—C6—C7	112.7 (5)	C22—N4—H4C	109.5
C5—C6—C7	125.7 (6)	H4A—N4—H4C	109.5
N1—C2—C3	121.4 (6)	H4B—N4—H4C	109.5
N1—C2—C1	111.8 (5)	C23—C24—H24A	109.5
C3—C2—C1	126.7 (6)	C23—C24—H24B	109.5
N2—C9—C10	121.9 (6)	H24A—C24—H24B	109.5
N2—C9—C8	112.5 (5)	C23—C24—H24C	109.5
C10—C9—C8	125.5 (6)	H24A—C24—H24C	109.5
O2—C1—O1	124.1 (6)	H24B—C24—H24C	109.5
O2—C1—C2	120.9 (5)	H15A—O15—H15B	83 (10)
O1—C1—C2	115.0 (5)		
			
O9—Zr1—O11—C21	15.8 (6)	O9—Zr1—N1—C2	38.3 (5)
O3—Zr1—O11—C21	−140.9 (5)	O3—Zr1—N1—C2	−179.9 (5)
O7—Zr1—O11—C21	−61.2 (5)	O7—Zr1—N1—C2	−139.5 (4)
O1—Zr1—O11—C21	82.2 (5)	O1—Zr1—N1—C2	−0.2 (4)
O5—Zr1—O11—C21	150.9 (4)	O5—Zr1—N1—C2	85.4 (4)
N3—Zr1—O11—C21	11.9 (4)	O11—Zr1—N1—C2	−96.1 (4)
N2—Zr1—O11—C21	−95.6 (5)	N3—Zr1—N1—C2	−49.0 (5)
N1—Zr1—O11—C21	148.7 (5)	N2—Zr1—N1—C2	131.2 (4)
O9—Zr1—O5—C8	−69.6 (4)	Zr1—O3—C7—O4	170.3 (4)
O3—Zr1—O5—C8	76.2 (4)	Zr1—O3—C7—C6	−6.6 (7)
O7—Zr1—O5—C8	7.7 (5)	Zr1—O9—C15—O10	171.2 (5)
O1—Zr1—O5—C8	−147.2 (5)	Zr1—O9—C15—C16	−8.1 (7)
O11—Zr1—O5—C8	141.2 (4)	C2—N1—C6—C5	−0.6 (9)
N3—Zr1—O5—C8	−102.2 (5)	Zr1—N1—C6—C5	179.3 (5)
N2—Zr1—O5—C8	4.9 (4)	C2—N1—C6—C7	177.0 (5)
N1—Zr1—O5—C8	143.4 (5)	Zr1—N1—C6—C7	−3.1 (6)
O3—Zr1—O9—C15	142.8 (4)	O4—C7—C6—N1	−171.2 (5)
O7—Zr1—O9—C15	72.6 (4)	O3—C7—C6—N1	5.9 (7)
O1—Zr1—O9—C15	−69.5 (4)	O4—C7—C6—C5	6.2 (9)
O5—Zr1—O9—C15	−151.4 (5)	O3—C7—C6—C5	−176.6 (6)
O11—Zr1—O9—C15	1.6 (5)	C6—N1—C2—C3	−1.8 (8)
N3—Zr1—O9—C15	5.4 (4)	Zr1—N1—C2—C3	178.3 (4)
N2—Zr1—O9—C15	138.4 (5)	C6—N1—C2—C1	178.6 (5)
N1—Zr1—O9—C15	−105.9 (5)	Zr1—N1—C2—C1	−1.3 (6)
O9—Zr1—O3—C7	137.0 (4)	C13—N2—C9—C10	−1.0 (9)
O7—Zr1—O3—C7	−148.3 (5)	Zr1—N2—C9—C10	177.4 (5)
O1—Zr1—O3—C7	3.5 (5)	C13—N2—C9—C8	−177.9 (5)
O5—Zr1—O3—C7	74.5 (4)	Zr1—N2—C9—C8	0.5 (6)
O11—Zr1—O3—C7	−70.0 (4)	Zr1—O1—C1—O2	176.4 (4)
N3—Zr1—O3—C7	−107.1 (4)	Zr1—O1—C1—C2	−3.6 (7)
N2—Zr1—O3—C7	141.3 (5)	N1—C2—C1—O2	−177.0 (5)
N1—Zr1—O3—C7	3.8 (4)	C3—C2—C1—O2	3.4 (9)
O9—Zr1—N2—C9	80.1 (4)	N1—C2—C1—O1	2.9 (7)
O3—Zr1—N2—C9	−97.0 (4)	C3—C2—C1—O1	−176.7 (6)
O7—Zr1—N2—C9	179.7 (5)	Zr1—O5—C8—O6	172.6 (4)
O1—Zr1—N2—C9	39.2 (5)	Zr1—O5—C8—C9	−6.3 (6)
O5—Zr1—N2—C9	−2.4 (4)	N2—C9—C8—O6	−175.6 (5)
O11—Zr1—N2—C9	−143.9 (4)	C10—C9—C8—O6	7.6 (9)
N3—Zr1—N2—C9	130.3 (4)	N2—C9—C8—O5	3.3 (7)
N1—Zr1—N2—C9	−49.9 (5)	C10—C9—C8—O5	−173.5 (6)
O9—Zr1—N2—C13	−101.4 (4)	Zr1—O7—C14—O8	176.7 (5)
O3—Zr1—N2—C13	81.4 (4)	Zr1—O7—C14—C13	−2.5 (7)
O7—Zr1—N2—C13	−1.9 (4)	C9—N2—C13—C12	0.3 (9)
O1—Zr1—N2—C13	−142.4 (4)	Zr1—N2—C13—C12	−178.1 (4)
O5—Zr1—N2—C13	176.0 (5)	C9—N2—C13—C14	179.8 (5)
O11—Zr1—N2—C13	34.5 (5)	Zr1—N2—C13—C14	1.4 (6)
N3—Zr1—N2—C13	−51.2 (5)	O8—C14—C13—N2	−178.7 (6)
N1—Zr1—N2—C13	128.5 (4)	O7—C14—C13—N2	0.5 (7)
O9—Zr1—O7—C14	70.8 (5)	O8—C14—C13—C12	0.8 (10)
O3—Zr1—O7—C14	−72.8 (5)	O7—C14—C13—C12	−180.0 (6)
O1—Zr1—O7—C14	139.0 (4)	C20—N3—C16—C17	0.3 (9)
O5—Zr1—O7—C14	−0.4 (6)	Zr1—N3—C16—C17	177.4 (5)
O11—Zr1—O7—C14	−152.5 (5)	C20—N3—C16—C15	−178.9 (5)
N3—Zr1—O7—C14	136.7 (5)	Zr1—N3—C16—C15	−1.8 (6)
N2—Zr1—O7—C14	2.4 (5)	O10—C15—C16—N3	−173.5 (5)
N1—Zr1—O7—C14	−110.7 (5)	O9—C15—C16—N3	5.9 (7)
O9—Zr1—O1—C1	−150.1 (5)	O10—C15—C16—C17	7.4 (9)
O3—Zr1—O1—C1	2.4 (6)	O9—C15—C16—C17	−173.2 (6)
O7—Zr1—O1—C1	136.3 (4)	C16—N3—C20—C19	0.4 (9)
O5—Zr1—O1—C1	−71.8 (5)	Zr1—N3—C20—C19	−176.6 (5)
O11—Zr1—O1—C1	71.7 (5)	C16—N3—C20—C21	−179.9 (5)
N3—Zr1—O1—C1	138.7 (5)	Zr1—N3—C20—C21	3.1 (7)
N2—Zr1—O1—C1	−110.4 (5)	Zr1—O11—C21—O12	164.3 (5)
N1—Zr1—O1—C1	2.1 (4)	Zr1—O11—C21—C20	−14.5 (7)
O9—Zr1—N3—C16	−1.3 (4)	N3—C20—C21—O12	−172.3 (6)
O3—Zr1—N3—C16	−144.6 (4)	C19—C20—C21—O12	7.4 (10)
O7—Zr1—N3—C16	−101.3 (4)	N3—C20—C21—O11	6.7 (8)
O1—Zr1—N3—C16	80.3 (4)	C19—C20—C21—O11	−173.7 (7)
O5—Zr1—N3—C16	33.0 (5)	N1—C2—C3—C4	2.7 (9)
O11—Zr1—N3—C16	175.8 (5)	C1—C2—C3—C4	−177.8 (6)
N2—Zr1—N3—C16	−52.7 (5)	C5—C4—C3—C2	−1.2 (11)
N1—Zr1—N3—C16	127.5 (4)	C3—C4—C5—C6	−1.0 (11)
O9—Zr1—N3—C20	175.7 (5)	N1—C6—C5—C4	1.9 (10)
O3—Zr1—N3—C20	32.4 (5)	C7—C6—C5—C4	−175.3 (6)
O7—Zr1—N3—C20	75.7 (4)	N3—C20—C19—C18	−0.1 (11)
O1—Zr1—N3—C20	−102.7 (5)	C21—C20—C19—C18	−179.7 (7)
O5—Zr1—N3—C20	−150.0 (4)	C17—C18—C19—C20	−1.0 (12)
O11—Zr1—N3—C20	−7.2 (4)	C10—C11—C12—C13	−0.3 (10)
N2—Zr1—N3—C20	124.3 (4)	N2—C13—C12—C11	0.4 (10)
N1—Zr1—N3—C20	−55.5 (5)	C14—C13—C12—C11	−179.1 (6)
O9—Zr1—N1—C6	−141.6 (4)	N3—C16—C17—C18	−1.4 (10)
O3—Zr1—N1—C6	0.2 (4)	C15—C16—C17—C18	177.6 (6)
O7—Zr1—N1—C6	40.6 (5)	C19—C18—C17—C16	1.8 (12)
O1—Zr1—N1—C6	179.9 (5)	C12—C11—C10—C9	−0.3 (10)
O5—Zr1—N1—C6	−94.5 (4)	N2—C9—C10—C11	1.0 (10)
O11—Zr1—N1—C6	84.0 (4)	C8—C9—C10—C11	177.5 (6)
N3—Zr1—N1—C6	131.1 (4)	N5—C23—C22—N4	162.0 (6)
N2—Zr1—N1—C6	−48.7 (5)	C24—C23—C22—N4	−77.5 (8)

### Hydrogen-bond geometry (Å, °)

**Table:**

*D*—H···*A*	*D*—H	H···*A*	*D*···*A*	*D*—H···*A*
O13—H13A···O14	0.80 (8)	2.10 (9)	2.745 (11)	138 (13)
O13—H13B···O2^i^	0.80 (4)	2.10 (8)	2.793 (8)	145 (12)
O14—H14A···O5^ii^	0.91 (8)	2.11 (9)	2.978 (7)	158 (12)
O14—H14B···O8	0.85 (4)	1.89 (5)	2.731 (9)	171 (14)
O15—H15A···O12	0.98 (9)	1.91 (10)	2.857 (8)	164 (12)
O15—H15B···O1^iii^	0.83 (4)	2.46 (7)	3.227 (9)	155 (13)
N4—H4A···O10^iv^	0.89	1.92	2.791 (8)	165.
N4—H4B···O6^ii^	0.89	2.24	2.851 (7)	125.
N4—H4C···O15^v^	0.89	2.04	2.864 (10)	154.
N5—H5A···O13	0.89	1.94	2.769 (9)	154.
N5—H5B···O6^iv^	0.89	2.18	2.844 (8)	131.
N5—H5C···O4	0.89	1.91	2.776 (8)	163.

Symmetry codes: (i) −*x*+3/2, *y*+1/2, −*z*+1/2; (ii) *x*−1/2, −*y*+3/2, *z*+1/2; (iii) −*x*+2, −*y*+1, −*z*+1; (iv) *x*−1, *y*, *z*; (v) −*x*+1, −*y*+1, −*z*+1.
